# 

**Published:** 1861-03

**Authors:** 


### CONTENTS.

### ORIGINAL ESSAYS AND COMMUNICATIONS.

Address to the Graduating Class, Ohio College of Dental Surgery—Session 1860-'61. By J. RICHARDSON, .......... 133
Reply. By A. EVANS, D. D. S., .......... 144
Dentition .......... 147
Dental Fees .......... 151
Who are Dentists. By WM. A. PEASE .......... 153
Refitting Temporary Plates. By J. RICHARDSON, .......... 156

### PROCEEDINGS OF SOCIETIES.

Michigan Dental Association. .......... 160
Central Ohio Dental Association .......... 165
Minutes of the Ninth Annual Meeting of the Ohio Dental College Association .......... 168
Minutes of the 17th Annual Meeting of the Miss. Valley Association of Dental Surgeons. 174

### SELECTIONS.

Causes which Modify the action of Anæsthetics—Dangers incident to Anæsthetics .......... 180
The Study of Medical Ethics, .......... 185
Extensive Medullary Cancer of the Antrum .......... 187

### CORRESPONDENCE.

A Letter from Philadelphia .......... 190
EDITORIAL. .......... 195

